# Newcastle Disease Virus as a Vaccine Vector for Development of Human and Veterinary Vaccines

**DOI:** 10.3390/v8070183

**Published:** 2016-07-04

**Authors:** Shin-Hee Kim, Siba K. Samal

**Affiliations:** Virginia-Maryland Regional College of Veterinary Medicine, University of Maryland, College Park, MD 20742, USA; shinkim@umd.edu

**Keywords:** Newcastle disease virus, vaccine vector, human vaccines, veterinary vaccines

## Abstract

Viral vaccine vectors have shown to be effective in inducing a robust immune response against the vaccine antigen. Newcastle disease virus (NDV), an avian paramyxovirus, is a promising vaccine vector against human and veterinary pathogens. Avirulent NDV strains LaSota and B1 have long track records of safety and efficacy. Therefore, use of these strains as vaccine vectors is highly safe in avian and non-avian species. NDV replicates efficiently in the respiratory track of the host and induces strong local and systemic immune responses against the foreign antigen. As a vaccine vector, NDV can accommodate foreign sequences with a good degree of stability and as a RNA virus, there is limited possibility for recombination with host cell DNA. Using NDV as a vaccine vector in humans offers several advantages over other viral vaccine vectors. NDV is safe in humans due to host range restriction and there is no pre-existing antibody to NDV in the human population. NDV is antigenically distinct from common human pathogens. NDV replicates to high titer in a cell line acceptable for human vaccine development. Therefore, NDV is an attractive vaccine vector for human pathogens for which vaccines are currently not available. NDV is also an attractive vaccine vector for animal pathogens.

## 1. Introduction

Infectious diseases have been emerging and reemerging over millennia [1]. Human immunodeficiency virus (HIV), severe acute respiratory syndrome coronavirus (SARS-CoV), and the most recent 2009 pandemic H1N1 influenza virus are only a few of many examples of emerging infectious pathogens in the modern world [2]. Each of these diseases has global societal and economic impact related to unexpected illnesses and deaths, as well as interference with travel, business, and daily activities. To overcome emerging, reemerging, as well as stable infectious diseases, the demand for development of efficient vaccines has greatly increased. Historically, live attenuated vaccines have provided the most effective protection against viral infection and disease [3]. However, there have been safety concerns with the risk of reversion to the wild-type pathogen phenotype as shown with some traditional live attenuated vaccines such as the polio vaccine. Furthermore, development of live attenuated vaccines has not been successful for many important pathogens. On the other hand, inactivated vaccines are generally not very effective and require a high containment laboratory for cultivation of highly virulent pathogens. Also, there is a risk of incomplete inactivation for inactivated vaccines. Therefore, there is a need for an alternative approach for development of vaccines. 

Replicating viral vector vaccines offer a live vaccine approach without requiring involvement of the complete pathogen or cultivation of the pathogen [4]. Replicating viral vectors have the ability to synthesize the foreign antigen intracellularly and induce humoral, cellular, and mucosal immune responses. Specifically, vectored vaccines can have advantages for (i) viruses for which a live attenuated vaccine might not be feasible (i.e., HIV); (ii) viruses that do not grow well in vitro (i.e., human papillomavirus, hepatitis C virus, and norovirus); (iii) highly pathogenic viruses that present safety challenges during vaccine development (i.e., SARS-CoV and Ebola virus); (iv) viruses that lose infectivity due to physical instability (i.e., respiratory syncytial virus (RSV)); and (v) viruses that can exchange genes with circulating viruses (i.e., coronaviruses, influenza viruses, and enteroviruses) [4]. A vectored vaccine can be rapidly engineered against a newly emerging pathogenic virus by inserting the gene of the protective antigen of the virus into the genome of the viral vector. In general, the magnitude of the immune response to live viral vector vaccines is substantially greater and broader than that induced by vaccines based on subunit proteins or inactivated viruses. Furthermore, manufacturing of vectored vaccines against highly pathogenic viruses do not require a high level of biosafety containment laboratories. 

Newcastle disease virus (NDV) is a fast-replicating avian virus that is prevalent in all species of birds [5]. In most avian species, NDV infections do not result in disease. In chickens, NDV causes a highly contagious respiratory and neurologic disease, leading to severe economic losses in the poultry industry worldwide [6]. NDV strains vary widely in virulence. Based on the severity of the disease in chickens, NDV strains are classified into three pathotypes: lentogenic strains which cause mild or asymptomatic infections that are restricted to the respiratory tract; mesogenic strains which are of intermediate virulence; and velogenic strains which cause systemic infections with high mortality [5]. Naturally occurring low-virulent NDV strains, such as LaSota and B1, are widely used as live attenuated vaccines to control Newcastle disease in poultry. Although NDV primarily infects avian species, many non-avian species have also been shown to be naturally or experimentally susceptible to infection. The advent of a reverse genetics system to manipulate the genome of NDV not only allowed us to study the molecular biology and pathogenesis of NDV but also to develop NDV as a vaccine vector against diseases of humans and animals. NDV vector has several advantages over other replicating viral vectors. 

Avirulent NDV strains are highly safe in avian and non-avian species. NDV replicates well in vivo and induces a robust immune response. In contrast to adeno, herpes, and pox virus vectors whose genome encodes a large number of proteins, NDV encodes only seven proteins and is thus less competition for immune responses between vector proteins and the expressed foreign antigen. NDV replicates in the cytoplasm, does not integrate into the host cell DNA, and does not establish persistent infection. Recombination involving NDV is extremely rare. NDV has a modular genome that facilitates genetic manipulation. NDV infects via the intranasal route and therefore induces both mucosal and systemic immune responses. A wide range of NDV strains exists that can be used as vaccine vectors. NDV-vectored vaccine can also be used as a “differentiating infected from vaccinated animals” (DIVA) vaccine. In this review article, we have reviewed the biology of NDV, development of reverse genetic systems for generation of NDV-vectored vaccines, and use of NDV vector for development of human and veterinary vaccines.

## 2. Biology of Newcastle Disease Virus (NDV) 

**Figure 1:**
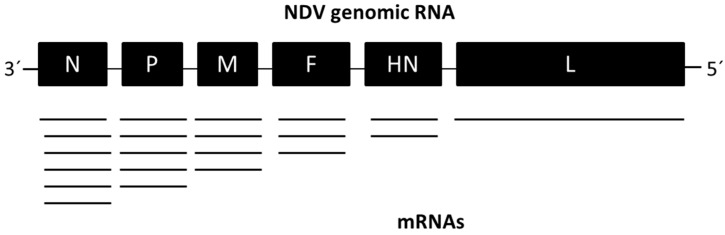
Genome organization and transcription scheme of Newcastle disease virus (NDV).

NDV is a member of the genus *Avulavirus* in the family *Paramyxoviridae* [5]. NDV virions are pleomorphic, but mostly spherical with a diameter of 100 nm. The virion is enveloped with a bilayer lipid membrane. The genome of NDV is a non-segmented, negative-sense, single-stranded RNA of 15,186 to 15,198 nucleotides containing six transcriptional units (3′-N-P-M-F-HN-L-5′) (Figure 1). The genome encodes a nucleocapsid protein (N), a phosphoprotein (P), a matrix protein (M), a fusion protein (F), a hemagglutinin-neuraminidase protein (HN), and a large polymerase protein (L). An additional protein called the V protein is produced by RNA editing of the P gene. The beginning and end of each gene contain control sequences, known as gene-start (GS) and gene-end (GE), respectively. The viral RNA-dependent RNA polymerase begins transcription at the 3′ end of the genomic RNA, in a sequential manner by a stop-start mechanism [5]. The re-initiation of transcription at the GS is not perfect, thus leading to a gradient of mRNA abundance with high levels of mRNA transcription located at the 3′ end. The genome length of NDV must be an even multiple of six for efficient virus replication following the “rule of six” [5]. 

In NDV, the HN and F proteins are the two integral membrane proteins. The HN protein is responsible for attachment of the virion to sialic acid containing cell surface receptors. The F protein mediates entry of the virus into the host cell by fusion of the viral envelope to the plasma membrane. The F protein is synthesized as a precursor (F0) that is cleaved by host cell protease into two biologically active F1 and F2 subunits. Cleavage of the F protein is a pre-requisite for virus entry and cell-to-cell fusion. The amino acid sequence at the F protein cleavage site has been identified as the primary determinant of virulence [7,8]. Virulent NDV strains have multibasic residues that conform to the preferred cleavage site of the intracellular protease furin present in most cell types. In contrast, avirulent NDV strains typically contain one or two basic residues at the F protein cleavage site and are delivered to the plasma membrane in an uncleaved form for cleavage by extracellular proteases, thus restricting viral replication to the respiratory and enteric tracts where secreted proteases for cleavage are available. 

## 3. Construction of NDV-Vectored Vaccines

**Figure 2:**
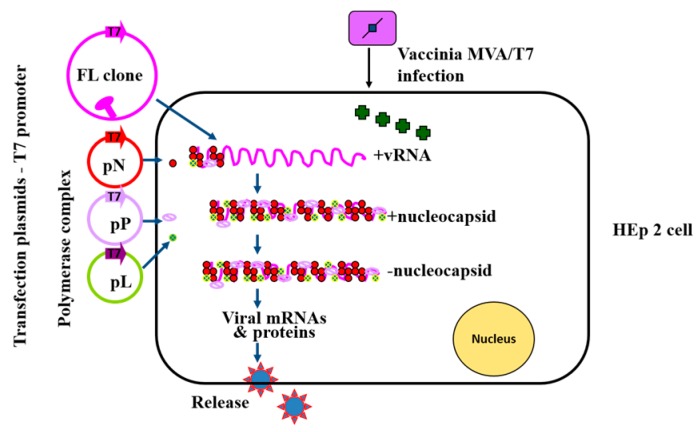
Plasmid-based recovery of recombinant NDV. HEp-2 cells are cotransfected with the antigenome plasmid and expression plasmids encoding the N, P and L proteins of NDV. The T7 RNA polymerase is provided by the recombinant vaccinia MVA/T7 strain.

Infectious NDV can be recovered entirely from cloned cDNA by transfecting cultured cells with plasmids encoding the viral components of a functional nucleocapsid, full-length antigenomic RNA, and the major proteins involved in replication and transcription, i.e., the N, P, and L proteins under the control of bacteriophage T7 RNA polymerase promoter [5] (Figure 2). This method, which is also known as reverse genetics technique, is now available for all three pathotypes of NDV strains [9,10,11,12]. In general, a foreign gene flanked by NDV GS and GE sequences is inserted into a 3′ non-coding region of an NDV genome as an additional transcription unit. Due to a polar gradient transcription, foreign genes are expressed more efficiently when placed closer to 3′ end of the genome. Although a foreign gene can be placed between any two genes of NDV, the insertion site between the P and M genes has been found optimal for efficient expression of the foreign protein and replication of NDV [13,14,15]. The insertion of a foreign gene into NDV genome increases its genome length and gene number and often has a growth retardation effect on virus replication in vitro and in vivo [16]. NDV accommodates foreign genes (at least 4.5 kb in length) with a good degree of stability [5]. A single NDV vector can also express at least three different foreign genes. 

Avirulent NDV strains LaSota and B1 are commonly used as vaccine vectors because of their proven track records of safety. Mesogenic and velogenic NDV strains are not used as vaccine vectors because they are virulent in chickens. In an experimental study, the mesogenic strain Beaudette C (BC) was evaluated as a vaccine vector in nonhuman primates [17]. Strain BC replicated to a higher titer and induced a substantially higher level of antibody response compared to strain LaSota, indicating it would be an effective vaccine vector. 

## 4. NDV as a Vaccine Vector for Human Use

NDV has several advantages for use as a vaccine vector in humans. NDV is safe in humans, due to a natural host range restriction. In nonhuman primates, the intranasal and intratracheal inoculation of African green and rhesus monkeys with 10^6.5^ plaque-forming units (PFU) per site of NDV did not cause any disease symptoms and its replication was restricted to the respiratory tract [17]. In humans, infection by NDV appears to be limited and benign based on both anecdotal observations with bird handlers and in clinical studies using NDV as an oncolytic agent [5]. According to a clinical study for NDV as an oncolytic agent in humans, intravenous administration of 10^10^ PFU of NDV to humans was safe without causing adverse effects [18]. NDV shares only a low level of amino acid sequence identity with known human paramyxoviruses and are antigenically distinct from common human and animal pathogens, and thus would not be affected by preexisting immunity in humans. NDV infects via the intranasal route and has been shown to induce humoral and cellular immune responses both at the mucosal and systemic levels in murine and nonhuman primate models [13,17,19,20,21,22,23,24,25,26]. NDV is a strong stimulator of the host immune response, thus providing an adjuvant effect. The use of avirulent pathotypes of NDV in humans prevents the possibility of accidental spread of a virulent virus strain from treated patients to birds. NDV grows to high titers not only in embryonated eggs (10^9^ PFU/mL) but also in Vero cells (10^8^ PFU/mL), which is acceptable for human vaccine development. In fact, NDV has been used to express protective antigens of various human pathogens and has shown promising results in nonhuman primates. NDV-vectored vaccines for several human pathogens are discussed as follows (Table 1).

The potential of recombinant NDV strain B1 as an effective vaccine vector for humans was first evaluated by expressing an influenza virus (A/WSN/33) hemagglutinin (HA) protein [13]. The expressed HA protein was incorporated into virions and appeared to be cleaved, indicating that the HA protein was accessible to proteolytic enzymes. In vitro growth kinetics and pathogenicity test in embryonated chicken eggs indicated attenuation of the recombinant NDV. Intravenous administration of mice induced higher titers of antibody to influenza virus HA than intraperitoneal administration. Further, immunized mice by the intravenous route were completely protected against a lethal dose of influenza virus, suggesting that NDV can be a safe and effective vaccine vector for possible use in mammalian and avian species.

The potential of NDV as a vaccine vector for use in humans was first determined in nonhuman primates. Two NDV strains LaSota and BC were evaluated as vaccine vectors in nonhuman primates by inserting the HN protein of human parainfluenza virus type 3 (HPIV3) as a protective antigen [17]. Two doses of immunization with NDV strains confirmed their restricted replication in African green monkeys (NDV-BC and NDV-LS) and in rhesus monkeys (NDV-BC only). However, the serum antibody response following the second dose exceeded that observed with HPIV3 infection, even though HPIV3 replicated much more efficiently than NDV in these animals. This is the first study to demonstrate efficacy of NDV-vectored vaccine in nonhuman primates.

Ebola virus (EBOV) causes severe hemorrhagic fever in humans with a fatality rate of up to 88% (species *Zaire* *ebolavirus*) of infected individuals [19]. Due to the limitation of inactivated vaccines, viral vectors based on common human pathogens have been used for EBOV vaccine. To overcome the high seroprevalence against vectors based on common human pathogens in the adult human population, recombinant NDV strain LaSota expressing the EBOV GP envelope protein was generated to evaluate its potential as a vaccine for EBOV [19]. Following one intranasal and intratracheal inoculation of rhesus monkeys with NDV/GP, titers of EBOV-specific antibodies and serum EBOV-neutralizing antibodies, were undetectable or low compared to those induced by HPIV3/GP. However, a second immunization resulted in a substantial boost in serum immunoglobulin (Ig) G enzyme-linked immunosorbent assay (ELISA) titers, yet the titers remained lower than those induced by a second dose of HPIV3/GP. In contrast, the ELISA IgA titers in respiratory tract secretions and the serum EBOV-neutralizing antibody titers were equal to those induced after the second dose of HPIV3/GP, showing that the efficacy of NDV vector can be comparable to that of HPIV3 vector by prime-boosting vaccination [23].

NDV was evaluated as a vaccine vector for another important emerging pathogen, the severe acute respiratory syndrome-associated coronavirus (SARS-CoV) [20]. Two NDV vectors were constructed: mesogenic strain BC (NDV-BC) and lentogenic strain LaSota in which the F protein cleavage sequence was modified to that of strain BC (NDV-VF) [20]. These NDV vectors were engineered to express the SARS-CoV spike S glycoprotein, the major protective antigen. Two dose immunizations of African green monkeys induced a robust neutralizing antibody response, resulting in reduction of virus shedding after challenge with SARS-CoV (10^6^ 50 % Tissue Culture Infective Dose (TCID_50_)). Specifically, immunization with NDV-VF vector resulted in SARS-CoV titers of a 5-fold, 61-fold, and 236-fold reduction in nasal turbinate, trachea, and lung, respectively, compared with the control animals. The NDV-BC vector was even more effective, with average reductions in viral titer of 13-fold, 276-fold, and 1102-fold in the nasal turbinate, trachea, and lung, respectively. This study demonstrated the safety and protective efficacy of NDV as a topical respiratory vaccine vector for SARS-CoV.

The use of viral vectors expressing selected HIV antigens has been a promising vaccine strategy. The potential of NDV-vectored vaccine against HIV infection was first evaluated by generating recombinant NDV expressing simian immunodeficiency virus (SIV) Gag protein (rNDV/SIVgag) [27]. The vaccine virus induced Gag-specific cellular immune responses in mice. Among intravenous, intraperitoneal, and intranasal immunization routes, intranasal administration induced the strongest protective immune response against a surrogate challenge virus (rVac/SIVgag) following a booster immunization with recombinant influenza viruses expressing immunogenic portions of SIV Gag. Specifically, this heterologous vaccination approach resulted in approximately, a 10^6^-fold reduction in rVac/SIVgag titers at day 5 after challenge compared to titers of control mice injected with phosphate-buffered saline (PBS). The magnitude of the protective immune response also correlated with the levels of cellular immune responses to Gag. These results suggest that NDV vector can be a suitable candidate vaccine against HIV.

The HIV Gag and Env proteins have been expressed by NDV vector [14,28,29,30]. The expression level of Gag protein was optimized using different insertion sites in the NDV genome. It was found that the codon-optimized Gag inserted between the P and M genes of NDV induced the highest level of protein expression and an enhanced immune response against HIV Gag in mice [14]. In another study, expression of gp160 Env protein by NDV vector LaSota also induced systemic and mucosal antibody responses in guinea pigs [28]. Priming/boosting by the intranasal route was more immunogenic than by the intramuscular route. Further, coexpression of gp160 Env and p55 Gag by vector LaSota enhanced both Env-specific and Gag-specific immune responses in guinea pigs [29]. This approach was efficient in inducing cellular and protective immune responses to challenge with vaccinia viruses expressing HIV-1 Env and Gag in mice. These results suggest that vaccination with a single NDV vector coexpressing Env and Gag represents a promising strategy to enhance immunogenicity and protective efficacy against HIV. In addition, heterologous prime (NDV expressing gp160) and boosting (purified gp120 protein) approach induced high neutralizing antibody titer in guinea pigs [30]. These findings suggest that vaccination with multiple HIV antigens in combination can broaden antiviral immune responses.

Respiratory syncytial virus (RSV) is a major cause of severe lower respiratory tract disease in infants and elderly [31]. The development of an effective vaccine against RSV is a high priority. In order to develop a vector vaccine against RSV, NDV strain B1 was used to express the fusion glycoprotein of RSV [31]. NDV was chosen as a viral vector because of its ability to induce a strong interferon (IFN)-α/β response. The RSV F protein was more immunogenic when presented by NDV-F than by live RSV, and this correlated with an increased ability of NDV to activate antigen-presenting cells in vitro and to induce high levels of IFN-α/β in vivo. RSV F-specific, CD8+ memory T cells were present in greater numbers in NDV-F-primed BALB/c mice than in animals previously infected with RSV. Consequently, NDV vaccine virus provided protection from RSV challenge. This study also highlights the adjuvant effect of NDV vector mediated by the potent IFN induction.

NDV has also been used as a vector to express the immunogens of a bacterial pathogen. Lyme borreliosis is a prevalent vector-borne disease in the United States, Europe and parts of Asia. NDV was used to express the basic membrane protein A (BmpA) and the outer surface protein C (OspC) of the Lyme disease pathogen *Borrelia burgdorferi* [33]. C3H or Balb/C mice that were immunized intranasally with the NDV vectors mounted vigorous serum antibody responses against the NDV vector, but failed to mount a robust response against either the intracellular or extracellular forms of BmpA or OspC. In contrast, a single immunization of hamsters with the NDV vectors via the intranasal, intramuscular, or intraperitoneal route resulted in rapid and rigorous antibody responses against the BmpA and OspC. Challenged with *B. burgdorferi* (10^8^ cells/animal), immunization with vector-expressing BmpA provided a reduction of the pathogen load in the joints. This study showed the potential of NDV as a vaccine vector against bacterial pathogens. 

Nipah virus (NiV) is a deadly emerging zoonotic pathogen that causes fatal encephalitis in humans and pigs [32]. The glycoprotein (G) and fusion protein (F) are two major NiV surface glycoproteins that stimulate protective immune responses. NDV strain LaSota expressing the NiV G and F proteins (rLa-NiVG and rLa-NiVF, respectively) were evaluated for their immunogenicity in mice and in pigs [32]. Following the second dose of immunization, rLa-NiVG and rLa-NiVF induced NiV-specific neutralizing antibodies in mice and long-lasting neutralizing antibodies in pigs (at least for 21 weeks). This study also showed that rLa-NiVG induced higher levels of neutralizing antibodies than rLa-NiVF. Although the protective efficacy of the vaccines was not evaluated in this study, the vaccine viruses showed the potential to be used for protecting humans and animals against NiV infection. 

Norovirus (NoV) is the most frequent cause of viral gastroenteritis in people of all ages [34]. The inability of NoV to grow in the cell culture system has greatly hindered development of effective vaccines. To circumvent this obstacle, virus-like particles (VLPs) produced by the baculovirus expression system have been commonly used as NoV vaccine candidates. As a live vaccine vector, LaSota and modified BC strains were used to express the capsid protein (VP1) of NoV strain VA387 (GII.4) and Norwalk virus (GI.1) [25,26]. For the modified BC vector, the multibasic cleavage site sequence of the F gene was changed to that of strain LaSota. The NoV-expressed VP1 protein formed VLPs in cell culture and in allantoic fluid of embryonated chicken eggs. The modified BC-vectored vaccine induced higher levels of serum, cellular, and mucosal immune responses than the baculovirus-expressed VLPs in mice. These results suggested that NDV has great potential for developing a live NoV vaccine. Alternatively, VLPs produced in large quantities in embryonated eggs or in cell culture by NDV can be a cost-effective method for producing a VLP-based vaccine for humans. This study also has implications for development of NDV-vectored vaccines for other non-cultivable pathogens of humans. 

## 5. NDV as a Vaccine Vector for Veterinary Use

NDV-vectored vaccines have been evaluated in several animal species (i.e., chicken, cattle, sheep, cat, mouse, pig, and dog) for veterinary use [35] (Table 2). NDV is a natural vaccine vector for poultry pathogens. Live attenuated NDV vaccines are widely used all over the world. Therefore, an NDV vector carrying the protective antigen of another avian pathogen can be used as a bivalent vaccine. Such a vaccine will be economical for poultry farmers. 

As a bivalent vaccine, the NDV strain LaSota was first used to express the host-protective immunogen VP2 of infectious bursal disease virus (IBDV), a birnavirus, which causes a highly immunosuppressive disease in chickens [36]. The protective efficacy of LaSota-expressing VP2 protein was evaluated by challenging vaccinated chickens with a highly virulent NDV strain Texas GB or a virulent IBDV variant strain. Vaccination with rLaSota/VP2 provided 90% protection against NDV and IBDV. Booster immunization induced higher levels of antibody responses against both NDV and IBDV and conferred complete protection against both viruses. These results indicate that the recombinant NDV can be used as a vaccine vector for other avian pathogens. 

Infectious laryngotracheitis is a major respiratory disease in chickens and caused by infectious laryngotracheitis virus (ILTV), a herpes virus [47]. Bivalent NDV-vectored vaccines against ILTV have been developed to improve the safety of current live attenuated ILTV vaccines [38,48]. The protective efficacy of NDVs expressing the three major ILTV surface glycoproteins, namely, gB, gC, and gD was evaluated against ILTV infection in chickens [38]. Particularly, rNDV-expressing gD induced the highest level of neutralizing antibodies among the tested vaccine candidates and completely protected chickens against the challenge of virulent ILTV and NDV, showing its potential as a bivalent vaccine. This protective efficacy of rNDV gD vaccine was attributed to high levels of envelope incorporation and cell surface expression of gD compared to gB and gC. In another study, LaSota viruses expressing gB and gD of ILTV were generated and vaccination of chickens with the two viruses conferred protection against virulent ILTV and NDV challenges [48]. In addition, LaSota with gB showed the protection of commercial broilers against clinical disease. Discrepancy of vaccine efficacy between these two studies could be due to the different levels of gB and gD expressions by NDV vectors and experimental conditions.

Infectious bronchitis virus (IBV), a coronavirus, is an important avian pathogen, causing respiratory disease in broilers and poor egg production in breeders and layers worldwide [49]. The spike polypeptide S2 gene was expressed by LaSota (rLS/IBV.S2) [39]. The vaccine virus effectively elicited hemagglutination inhibition antibodies against NDV and protected chickens against lethal challenge with virulent strain NDV/CA02. IBV heterotypic protection was assessed using a prime-boost approach with a commercially available attenuated IBV Massachusetts (Mass)-type vaccine. Chickens primed ocularly with rLS/IBV.S2 and boosted with Mass were completely protected against challenge with a virulent Ark-type strain. The protective efficacy of this heterologous vaccination was similar to that of priming and boosting with Mass (Mass + Mass). Based on clinical signs, both vaccinated groups appeared equally protected against challenge compared to unvaccinated challenged chickens. In shedding of challenge virus in the trachea, viral RNA was detected in 50% of rLS/IBV.S2 + Mass-vaccinated chickens while chickens vaccinated with Mass + Mass and unvaccinated challenged controls showed 84% and 90% incidence of IBV RNA detection, respectively. These results demonstrate the potential of NDV-vectored vaccine for IBV infection. 

NDV vector has also been used for the prevention of economically important livestock diseases. Rift Valley fever virus (RVFV), a bunyavirus, causes recurrent large outbreaks in humans and in livestock [50]. NDV expressing the RVFV structural glycoproteins Gn was generated (NDFL-Gn) [40]. Immunization of calves via the intranasal route elicited no detectable antibody responses, whereas intramuscular immunization elicited antibodies against both NDV and the Gn protein. In general, the titers of RVFV-neutralizing antibodies were modest, varying from 8 to 32. To improve the efficacy of NDV-vectored vaccine, Gn was coexpressed with another glycoprotein Gc [41], which resulted in the formation of VLPs and subsequent release from the producing cells. A homologous prime-boost vaccination of mice with this vaccine virus induced neutralizing antibodies and provided complete protection from a lethal RVFV challenge. The immunogenicity of the vaccine virus was further evaluated in lamb, the main target species of RVFV. A single intramuscular vaccination induced neutralizing antibodies, and this response was significantly boosted by a second vaccination. Although coexpression of the Gn and Gc induced a good immune response, protective efficacy of this vaccine needs to be further evaluated.

Bovine herpesvirus-1 (BHV-1) is a major cause of respiratory tract diseases in cattle. Since modified live BHV-1 vaccines can cause latent infection in immunized animals, NDV expressing the glycoprotein D (gD) of BHV-1 was generated as a vectored vaccine [42]. A single intranasal and intratracheal inoculation of calves with NDV elicited mucosal and systemic antibodies specific to BHV-1. Challenge with BHV-1 showed reduced virus shedding and clinical signs in immunized calves compared to unimmunized claves. In addition, the titers of serum antibodies specific to BHV-1 were higher in immunized animals compared to unimmunized animals, indicating that the vaccines primed for secondary responses. This indicates that NDV can be used as a vaccine vector in bovines, and BHV-1 gD may be useful as a mucosal vaccine against BHV-1 infection. However, vaccination might require augmentation by a second dose or the inclusion of additional BHV-1 antigens.

Rabies virus (RV), a rhabdovirus, causes a fatal neurologic disease in humans and in animals [43]. To generate an effective, safe, and affordable rabies vaccine, NDV strain LaSota expressing the rabies virus glycoprotein G (rL-RVG) was evaluated. The safety of rL-RVG vaccine virus was confirmed in cats and dogs. Intramuscular vaccination with rL-RVG induced strong and long-lasting protective neutralization antibody responses against rabies virus in dogs and cats. Although three doses of vaccination were conducted, the second dose induced the highest levels of immune responses in both cats and dogs. Vaccination dose of 10 50% embryo infective dose (EID) completely protected dogs from challenge after one year. This study demonstrated protective efficacy of NDV-vectored vaccine against rabies in dogs. This vaccine may also have potential use in high-risk human individuals to control rabies virus infections.

Canine distemper virus (CDV), a morbillivirus, infects many carnivores and cause several high-mortality disease outbreaks [37]. The current CDV live vaccine cannot be safely used in some exotic species, such as mink and ferret. NDV strain LaSota expressing envelope glycoproteins, hemagglutinin (H, rLa-CDVH) and fusion protein (F, rLa-CDVF), were generated as vaccine candidates. In immunized minks, rLa-CDVH induced higher titers of neutralization antibodies against CDV than rLa-CDVF neutralizing antibodies. Further, rLa-CDVH provided complete protection against virulent CDV challenge during the four weeks of observation. In contrast, all animals immunized with rLa-CDVF developed clinical signs of distemper and virus shedding. This study suggested that recombinant NDV expressing the H protein of CDV is a safe and efficient candidate vaccine against CDV in mink. The efficacy of rLa-CDVH virus also needs to be evaluated in other host carnivore species.

## 6. NDV-Vectored Vaccines against Highly Pathogenic Avian Influenza Virus

Highly pathogenic avian influenza virus (HPAIV) is an economically important pathogen of poultry worldwide. The outbreaks involving H5N1 or H7N7 influenza viruses resulted in lethal infections in poultry and the death of a limited number of people [51]. Therefore, vaccination of poultry against HPAIV could play an important role in reducing virus shedding and raising the threshold for infection and transmission [46]. However, development of vaccines against HPAIV has been hampered due to poor immunogenicity of the virus [52]. Furthermore, inactivated vaccines are not commonly used because of the high cost due to the requirement of enhanced biosafety level 3 containment and the difficulty in “differentiating infected from vaccinated animals” (DIVA). The use of live attenuated influenza viruses as vaccines in avian or mammalian species can also raise a major biosafety concern, because the vaccine viruses may become virulent through mutation or genetic reassortment with circulating strains. Alternatively, NDV can be an ideal vaccine vector for development of an avian influenza vaccine. NDV infects via the intranasal route and therefore induces both local and systemic immune responses at the respiratory tract [5]. Therefore, it provides a convenient platform for rapid, efficient, and economical immunization. In fact, NDV has been most commonly used as a vaccine vector against AIV. Protective efficacy of NDV-vectored vaccines has been evaluated and verified by many different vaccination studies [21,22,44,45,46,53,54]. 

For the generation of vaccines, a major protective antigen, hemagglutinin (HA) of HPAIV has been placed between the P and M genes or between the F and HN genes in lentogenic NDV strains LaSota or B1. To address a safety concern, an NDV-vectored vaccine was further generated by replacing the polybasic cleavage site in HPAIV HA with that from a low-pathogenicity strain of influenza virus [22]. In addition, the HA gene has been modified to enhance its expression levels by NDV. Specifically, elimination of an NDV transcription termination signal-like sequence located within the HA open reading frame of H5 enhanced expression levels of HA protein by NDV and completely protected chickens after challenge with a lethal dose of velogenic NDV or highly pathogenic AIV, respectively [44]. In addition, the ectodomain of an H7N7 or H5N1 avian influenza virus HA was fused with the transmembrane and cytoplasmic domains derived from the F protein of NDV [46,53]. This approach resulted in enhanced incorporation of the foreign protein into virus particles and the protection of chickens against both HPAIV and a highly virulent NDV. These studies also demonstrated that NDV can be used to generate a bivalent vaccine. 

Although use of avirulent NDV vectors has been effective in protecting chickens against clinical disease and mortality, some studies also found virus shedding in chickens after challenge with HPAIV [45]. To enhance the replication of vaccine virus, attenuated mesogenic NDV strain BC has been generated by changing the multibasic cleavage site sequence of the F protein to the dibasic sequence of strain LaSota [54]. Additionally, the BC, F, and HN proteins were modified in several ways to enhance virus replication. The modified BC-based vectors replicated better than LaSota vector, and expressed higher levels of HA protein and provided complete protection against challenge virus shedding, suggesting its potential to be safely used as a vaccine vector. 

For effective human vaccines against HPAIV, the immunogenicity of NDV expressing the HA of H5N1 was evaluated in African green monkeys by the intranasal route of administration [21]. Two doses of NDV-vectored vaccine (2 × 10^7^ PFU) induced a high titer of H5N1 HPAIV-neutralizing serum antibodies in all of the immunized monkeys. Moreover, a substantial mucosal IgA response was induced in the respiratory tract, which can potentially reduce or prevent transmission of the virus during an outbreak or a pandemic. The intranasal route of administration is also advantageous for needle-free immunization and is thus suitable for mass immunization. The protective efficacy of vaccine viruses was evaluated in African green monkeys by the intranasal/intratracheal route or by the aerosol route of administration [22]. Each of the vaccine constructs was highly restricted for replication, with only low levels of virus shedding detected in respiratory secretions. All groups developed high levels of neutralizing antibodies against homologous (A/Vietnam/1203/04) and heterologous (A/egret/Egypt/1162-NAMRU3/06) strains of HPAIV and were protected against challenge with 2 × 10^7^ PFU of homologous HPAIV. This study demonstrated that needle-free, highly attenuated NDV-vectored vaccines were immunogenic and protective in a nonhuman primate model of HPAIV infection. 

## 7. Conclusions

Newcastle disease virus (NDV) is an attractive vaccine vector for both human and animal pathogens. The live attenuated vaccine strains used as vaccine vectors have a proven track record of safety and efficacy. NDV vectors not only induce robust humoral and cellular immune responses but also induce mucosal immune response. Therefore, NDV can be a vector of choice for mucosal immunization. The ability of NDV to infect a wide variety of non-avian species makes it a potential vector for other animals. NDV is also a promising vaccine vector for use in humans. One advantage is that most humans do not have pre-existing immunity to NDV. NDV-vectored vaccines have also become available commercially (i.e., H5N1 HPAIV vaccine for poultry).

## Figures and Tables

**Table 1:** NDV-vectored vaccines against human pathogens.

Pathogen	Disease	Antigen	NDV Strain	Animal Model	Route of Inoculation *	Dose	Ref.
Influenza A H1N1	Respiratory	HA	B1	Mouse	iv, ip	3 × 10^7^ PFU; two doses	[13]
HPAIV H5N1	Respiratory	HA	LaSota	African green monkey	in	2 × 10^7^ PFU; two doses	[21]
HPIV3	Respiratory	HN	LaSota BC	African green and rhesus monkeys	in, it	10^6.5^ PFU; two doses	[17]
EBOV	Hemorrhagic fever	GP	LaSota	Rhesus monkeys	in, it	Two doses	[19]
SARS-CoV	Respiratory	S	LaSota	African green monkeys	in, it	Two doses	[20]
SIV	simian AIDS	Gag	B1	Mouse	iv, ip, in	5 × 10^7^ PFU; one or two doses	[27]
HIV	AIDS	Gag	B1	Mouse	in	5 × 10^5^ PFU; two doses	[14]
HIV	AIDS	Env	LaSota	Guinea pig	in, im	10^6^ PFU; two or three doses	[28]
HIV	AIDS	Env Gag	LaSota	Guinea pig	in	10^6^ PFU; two or three doses	[29]
HIV	AIDS	Env (gp160, gp120)	LaSota	Guinea pig	in	10^6^ PFU; three doses	[30]
HRSV	Respiratory	F	B1	Mouse	in	10^5.5^ PFU; one dose	[31]
NiV	Encephalitis	F/G	LaSota	Mouse Pig	im	10^9^ EID_50_; two doses	[32]
Borrelia burgdorferi	Lyme	BmpA OspC	LaSota	Hamster	in, im, ip	10^6^ PFU	[33]
NoV	Gastroenteritis	VP1	LaSota BC	Mouse	in	10^6^ EID_50_; two doses	[25]

* im: intramuscular, in: intranasal, ip: intraperitoneal, it: intratracheal, iv: intravenous, EID_50_: 50% embryo infective dose.

**Table 2:** NDV-vectored vaccines against veterinary pathogens.

Pathogen	Disease	Antigen	NDV Strain	Animal Model	Route of Inoculation *	Dose	Ref.
IBDV	Immunosuppressive disease	VP2	LaSota	Chicken	o	10^4^ EID_50_; two doses	[36]
CDV	Canine distemper	F/HN	LaSota	Mink	im	10^9^ EID_50_; two doses	[37]
ILTV	Respiratory	gB, gC, gD	LaSota	Chicken	on	10^6^ TCID_50_	[38]
IBV	Respiratory	S2	LaSota	Chicken	o	10^9.8^ EID_50_; two doses	[39]
RVFV	Rift Valley fever	Gn	LaSota	Calf	im	10^7^ TCID_50_, two doses	[40]
RVFV	Rift Valley fever	Gn, Gc	LaSota	Mouse; Lamb	im	10^5.3^TCID_50_; 10^7.3^ TCID_50_; two doses	[41]
BHV-1	Respiratory	gD	LaSota	Calf	in	10^6^ PFU; one dose	[42]
RV	Rabies	G	LaSota	Cat; Dog	im	10^9.8^ EID_50_; 10^8.3^ EID_50_; three doses	[43]
HPAIV H5N1	Respiratory	HA	LaSota	Chicken	in	10^6^ EID_50_; one dose	[44,45]
HPAIV H7N2	Respiratory	HA	B1	Chicken	o	10^6^ EID_50_; one dose	[46]

* o: ocular, on: oculonasal, im: intramuscular, in: intranasal, it: intratracheal, EID_50_: 50% embryo infectious dose, TCID_50_: 50% tissue culture infective dose.
